# Neural dynamics of robust legged robots

**DOI:** 10.3389/frobt.2024.1324404

**Published:** 2024-04-18

**Authors:** Eugene R. Rush, Christoffer Heckman, Kaushik Jayaram, J. Sean Humbert

**Affiliations:** ^1^ Department of Mechanical Engineering, University of Colorado Boulder, Boulder, CO, United States; ^2^ Department of Computer Science, University of Colorado Boulder, Boulder, CO, United States

**Keywords:** robotics, locomotion, robustness, neuroscience, reinforcement learning

## Abstract

Legged robot control has improved in recent years with the rise of deep reinforcement learning, however, much of the underlying neural mechanisms remain difficult to interpret. Our aim is to leverage bio-inspired methods from computational neuroscience to better understand the neural activity of robust robot locomotion controllers. Similar to past work, we observe that terrain-based curriculum learning improves agent stability. We study the biomechanical responses and neural activity within our neural network controller by simultaneously pairing physical disturbances with targeted neural ablations. We identify an agile hip reflex that enables the robot to regain its balance and recover from lateral perturbations. Model gradients are employed to quantify the relative degree that various sensory feedback channels drive this reflexive behavior. We also find recurrent dynamics are implicated in robust behavior, and utilize sampling-based ablation methods to identify these key neurons. Our framework combines model-based and sampling-based methods for drawing causal relationships between neural network activity and robust embodied robot behavior.

## 1 Introduction

In recent years, embodied deep reinforcement learning (RL) systems have demonstrated intelligent behavior in real-world settings, especially in the areas of quadrupedal (Lee et al., 2020; Rudin et al., 2022a; Rudin et al., 2022b; Feng et al., 2022; Miki et al., 2022; Vollenweider et al., 2022) and bipedal locomotion (Siekmann et al., 2020; 2021). These accomplishments represent significant steps towards generating rich robot behavior that rivals their biological counterparts (Caluwaerts et al., 2023).

However, there remains a large gap in understanding the neural basis of these learning-based legged locomotion controllers, especially when it comes to stable, robust behavior. Some have examined locomotion robustness by varying the degree of controller decentralization during training (Schilling et al., 2020; 2021). Others have analyzed neural activity, though these efforts are relatively shallow. One example is identifying cyclic patterns of neural activity during walking (Siekmann et al., 2020), which is an unsurprising result given that locomotion is inherently a cyclic behavior. Another effort draws a connection between sensorimotor processing and a foot-trapping reflex behavior, but does not attempt to analyze the neural basis of this behavior (Lee et al., 2020).

The lack of research on interpretability of learned locomotion controllers may be due to the fact that most robotics and AI researchers focus more on functionality and performance and less on mechanistic understanding (Chance et al., 2020). In recent decades, however, there has been advances made in explainable artificial intelligence (XAI) (Kamath and Liu, 2021; Minh et al., 2022), which include reinforcement learning systems (Huber et al., 2019; Heuillet et al., 2021; Beechey et al., 2023; Hickling et al., 2023). A number of these methods quantify individual feature importance relative to model behavior (Bach et al., 2015; Samek et al., 2015; Lundberg and Lee, 2017; Shrikumar et al., 2019). These methods have been employed for a variety of RL tasks such as robot manipulation (Wang et al., 2020; Remman and Lekkas, 2021) and vehicle guidance (Liessner et al., 2021), however, not for locomotion control. Additionally, these studies do not consider disturbances and the feature importance that drives robust controller responses.

Despite the dearth of neural analysis efforts within learning-based legged robot control, there is a significant body of work inspired by computational neuroscience that studies task-oriented artificial neural networks (ANN) (Sussillo and Barak, 2013; Saxena and Cunningham, 2019; Vyas et al., 2020). Examples include training networks to perform tasks such as text classification (Aitken et al., 2022), sentiment analysis (Maheswaranathan et al., 2019a; Maheswaranathan and Sussillo, 2020), transitive inference (Kay et al., 2022), pose estimation (Cueva and Wei, 2018; Cueva et al., 2020b; 2021), memory (Cueva et al., 2020a), and more (Yang et al., 2019). Many of these studies employ recurrent neural networks (RNNs), which embed input information across time in latent neural states and process that information through latent neural dynamics. One advantage over biology, is the direct access to the full parameterization of these artificial models, which has enabled computational neuroscientists to begin reverse-engineering such systems.

The aforementioned tasks focus on open-loop estimation, where the system generating behavioral data is fixed, and does not interact with or involve feedback of the ANN output estimates. However, the widening application of deep reinforcement learning has allowed researchers to expand towards closed-loop control of embodied agents. In one recent study, researchers simulate a virtual rodent model and study neural activity across various high-level behaviors (Merel et al., 2020). Another *in silico* study (Singh et al., 2021) examines the population-level dynamics of a virtual insect localizing and navigating to the source of an odor plume. The analyses in both these studies reveal coordinated activity patterns in high-dimensional neural populations, however neither make direct connections to stability or robustness of legged locomotion. The former (Merel et al., 2020) focuses chiefly on features of multi-task neural behavior, and the latter (Singh et al., 2021) focuses on how neural activity relates to spatial localization and navigation.

In this work, we aim to explicitly investigate the neural basis of lateral stability of legged robots during walking. Prior work on embodied legged locomotion have conducted neural ablation studies to draw causal link between neural activity and behavior, yet no connection was made to walking stability (Merel et al., 2020). In this work, we draw inspiration from (Meyes et al., 2019; Towlson and Barabási, 2020), where ablations are extended from single neurons to pairs and triplets, as well as (Jonas and Kording, 2017), which suggests that lesioning studies could be more meaningful if we could simultaneously control the system at a given moment. Our method of controlling our agent is by applying precisely-timed surprise lateral perturbations, similar to those studied in animals (Karayannidou et al., 2009; Hof et al., 2010), and robots (Kasaei et al., 2023). However, our focus is not just on the bio-mechanical response, but on the neural activity that ultimately drives this stabilizing behavior.

The contributions of this paper include:

• Characterizing cyclic, low-dimensional neural activity of quadrupedal robot locomotion, which is consistent with prior neuroscience findings.
• Identifying key bio-mechanical responses implicated in robust recovery behavior to lateral perturbations, specifically a stepping strategy commonly found in legged animals.
• Elucidating the neural basis of robust locomotion through model-based and sampling-based ablation strategies.

## 2 Methods

We outline our methodology in training quadrupedal robotic agents to walk in a virtual physics simulator, using deep reinforcement learning. We provide details regarding the agent and environment, as well as model training and architecture. Given an RNN-based controller and its embodied motor control behavior, we discuss methods employed to elucidate the neural activity that enables the agent’s ability to reject disturbances.

### 2.1 Agent and environment

In this work, we utilize NVIDIA Isaac Gym (Makoviychuk et al., 2021), an end-to-end GPU-accelerated physics simulation environment, and a virtual model of the quadrupedal Anymal robot (Hutter et al., 2016; Rudin et al., 2022b) from IsaacGymEnvs^1^. The agent’s action space is continuous and consists of 12 motor torque commands, three for each leg. The agent’s proprioceptive observation space consists of 36 signals: three translational body velocities (*u*, *v*, *w*) representing longitudinal, lateral, and vertical body velocities, three rotational body velocities (*p*, *q*, *r*) representing roll rate, pitch rate, and yaw rate, orientation signals *sin*(*θ*), *sin*(*ϕ*), and $-\sqrt{1-sin^{2}\left(\theta \right)-sin^{2}\left(\phi \right)}$ , three planar body velocity commands (u*, v*, r*), 12-dimensional joint angle positions ***θ***_**joint**_, 12-dimensional joint angular velocity ***ω***_**joint**_. The agent additionally receives 140 exteroceptive observations in the form of depth measurements, ***d***, which are uniformly sampled from a 1 m × 1.6 m grid beneath the agent. These command and observation signals are computed directly from the physics simulation of the agent and environment. They are updated every control time step of 20 ms, and are simulated with uniformly distributed white noise. Note that there is a decimation of four simulation steps per control step, meaning the simulation time step is 5 ms, and there are four simulation time steps for every control time step.

### 2.2 Model training

Unlike conventional controllers, these deep RL-based controllers do not explicitly compute errors from command and feedback signals. Instead a policy is trained on a reward function that consists of a weighted sum of linear body velocity error ${\left(u^{*}-u\right)}^{2}+{\left(v^{*}-v\right)}^{2}$ and angular body velocity error $p^{2}+q^{2}+{\left(r^{*}-r\right)}^{2}$ , along with a suite of knee collision, joint acceleration, change in torque, and foot airtime penalties, similar to (Rudin et al., 2022b). During training, inputs to the agents include sensory signals, randomly generated linear body velocity commands (u*, v*), and an angular velocity command r* that is modulated to regulate to a random goal heading. Therefore, these command signals drive behavior indirectly through the reward function. This summarizes the training of the Baseline policy. During evaluation, the user specifies these command velocities based on the experiment. Most experiments in this work focus on forward walking, where v* = 0 m/s and r* = 0 rad/s.

To build on the Baseline policy, we generate a Terrain policy by employing a game-inspired curriculum, where agents are first trained on less challenging terrain before progressively increasing their complexity Rudin et al. (2022b). The environment is composed of a grid of sub-regions, where in one direction, the type of terrain varies from smooth slopes (10%), rough terrain (10%), stairs up (35%), stairs down (25%), and discrete terrain (20%), and in the other direction the difficulty of the terrain increases. Agents that successfully complete the sub-region they start in are moved to the next terrain level of difficulty, and agents that complete the hardest level are cycled back to a random terrain level to avoid catastrophic forgetting. Once agents can consistently complete the hardest level of terrain, agents become uniformly distributed across all terrain levels, rather than biased toward easier levels. For reference, the hardest level consists of 20° slopes and 0.20 m stair steps.

We expand upon Baseline and Terrain policies by introducing random force disturbances during training, which produce Disturbance and Terrain-Disturbance policies, respectively. Note that we are applying random external force perturbations, whereas Rudin et al. (2022b) applied random velocity perturbations. We choose to apply random external forces, since this allows the simulator to model the physical dynamics of the agent.

Disturbances are instantiated at random, with a 1% chance of a perturbation being initiated at each control time step. Once perturbations are instantiated, there is a 2% chance of termination each time step. This results in the duration of random perturbations following an exponential distribution. Each time a new perturbation is instantiate, its *x*, *y*, and *z*-direction force components are randomly sampled from a uniform distribution between −0.24 and 0.24 times body weight, and are held constant throughout the duration of the perturbation.

For all policies, agents are also exposed to sensory noise, as well as slight randomizations to gravity and friction during training. Training hyperparameters are listed in Table 1. Training of the Terrain-Disturbance policy took 10 h and 47 min on a NVIDIA GeForce RTX 2080 Ti.

**TABLE 1:** Hyperparameters.

Name	Value
Learning Rate	0.0003
Horizon Length	16
Mini-Batch Size	16,384
Mini-Epoch Size	4
Number of Environments	4,096
RNN BPPT Truncation Length	16
PPO Discount Factor Gamma	0.99
PPO Clipping Epsilon	0.2
PPO KL Threshold	0.008
PPO GAE Labmda	0.95
PPO Entropy Coefficient	0

### 2.3 Model architecture

**FIGURE 1:**
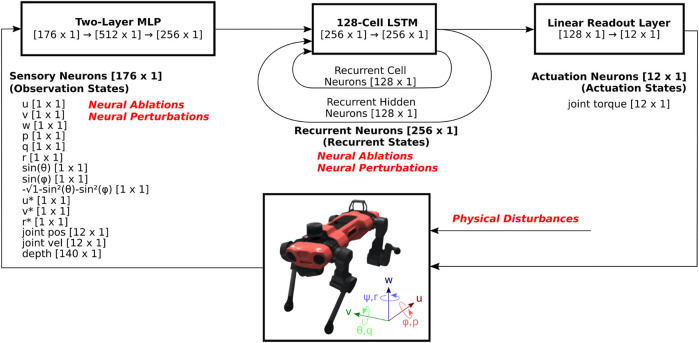
System diagram of RNN-based reinforcement learning locomotion controller. The observation states consists of body velocity commands and sensory feedback signals, which pass through an MLP and an RNN, in this case an LSTM network. The recurrent state feeds back on itself in the RNN, and part of it feeds into a fully-connected (FC) layer, and outputs the actuation state, which consists of 12 motor torque commands. As the output of the controller, these commands are sent to Anymal, which is modeled in the NVIDIA Isaac Gym physics simulator. The resulting observation states consist of sensory feedback signals such as body velocities, orientations, joint positions and velocities, as well as body velocity command signals. These are then updated and are fed back into the RL controller for the subsequent time step.

Agents are trained using a high-performance, open-source RL implementation, rl_games^2^, which implements a variant of Proximal Policy Optimization (PPO) (Schulman et al., 2017) that utilizes asymmetric inputs to actor and critic networks (Pinto et al., 2017). We utilize an implementation that integrates Long-Short Term Memory (LSTM) networks into both the actor and critic networks. Both actor and critic networks pass the observation vector [176 × 1] through dedicated multi-layer perceptrons (MLP) with two dense layers of size 512 and 256, a single-layer 128-cell LSTM network, and a fully-connected output layer that results in an action vector [12 × 1] as shown in Figure 1. Each LSTM unit consists of a cell state and a hidden state, which are capable of encoding long-term and short-term memory, respectively. Neural activations of these cell states [128 × 1] and hidden states [128 × 1] are collectively referred to as the recurrent states [256 × 1]. The action vector contains the motor torque commands for each of the 12 joints, and is referred to as the actuation state. The critic network is not illustrated, but has independently trained weights and identical structure, except that the fully-connected layer outputs a scalar value estimate, as opposed to as 12-dimensional actuation state.

### 2.4 Neural perturbations

We apply neural and physical perturbations during walking, with the aim of deepening our understanding of disturbance rejection properties of our locomotion controller during nominal operation. We draw inspiration for experiment design from primate studies, which found low-dimensional structure in their population dynamics (O’Shea et al., 2022), and to date has not been applied to RL-based agents. The only similar RL study (Merel et al., 2020) perturbed RNN hidden states by inactivating neuronal states or replacing with neural states from other behavioral policies, but did not apply targeted perturbations to better understand low-dimensional structure. For neural perturbations, we compute the population response in the top principal component (PC) directions, and in separate experiments, apply normal and tangential perturbations to the recurrent state during walking.

### 2.5 Physical perturbations

After training, we perform physical perturbations trials, where an external lateral force is applied to the robot at its center of mass for 80 ms, which is a similar duration as found in other works (Hof et al., 2010; Kasaei et al., 2023). Unless otherwise specified, we apply a perturbation of 3.5 times body weight at the moment when the LF and RH foot hit the ground. We then analyze the resulting sensory, recurrent, and actuation neural activity, as well as the bio-mechanical response. Our primary metric for stability is recovery rate, which is the percentage of trials in which agents successfully recover from the lateral perturbation, from 0% to 100%.

### 2.6 Dimensionality reduction

It can often be difficult with high-dimensional multivariate datasets to isolate and visualize lower-dimensional patterns, due to feature redundancy. Because of this, we perform principal component analysis (PCA), a linear dimensionaility reduction technique, to identify the directions of dominant activity in our neural populations. To do this, we utilize the scikit-learn Python library, first applying Z-scale normalization, and then applying PCA to the normalized data. This feature scaling through normalization, involves rescaling each feature such that it has a mean of 0 and standard deviation of 1. When presenting PC data, we constrain the data to the context in which it is presented. For example, when presenting observation states, recurrent states, and actuation states for various walking speeds, we found the principal components for each of those three datasets, with each dataset incorporating data across all walking speeds trials.

After training and during data collection rollouts, the agent is commanded with a range of forward speed commands u* between 0.8 and 2.0 m/s, while v* and r* remain at 0 m/s. We perform principal component analysis (PCA) on this dataset, in order to determine the dominant neural populations and improve interpretability. For all perturbation studies presented in this work, we hold the forward speed command u* at 2.0 m/s, and transform the data based on the original non-perturbed PCA transformation. During data collection, noise parameters and perturbations are removed, unless otherwise stated.

### 2.7 Subspace overlap

Subspace overlap is a quantity that measures the degree to which a population response occupies similar neural state space to another population response. This measure is utilized in comparing cyclic neural trajectories in primates (Russo et al., 2020), and is utilized similarly for robots in this work. The reference population response *R*_*A*_ is dimensions [*n* × *t*], where *n* is the number of neurons and *t* is the number of neural recordings. After applying PCA to *R*_*A*_, it yields *W*_*A*_ which has dimensions [*n* × *k*], where *k* is the number of PCs. Variance is computed as *V*(*R*, *W*) = 1 − |*R* − *RWW* ^*T*^|_*F*_/|*R*|_*F*_, where |⋅|_*F*_ is the Frobenius norm. The subspace overlap is computed as *V*(*R*_*B*_, *W*_*A*_)/*V*(*R*_*B*_, *W*_*B*_), and lower values indicate the neural populations occupy different neural dimensions, relative to one another.

### 2.8 Neural ablations

**FIGURE 5:**
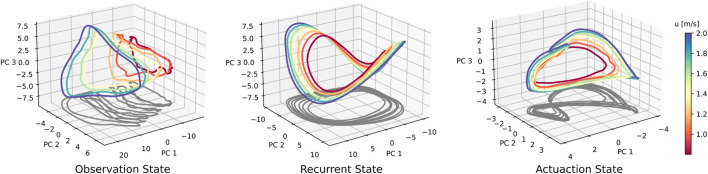
Neural data for a suite of forward speed commands, ranging from 0.8 to 2.0 m/s. Across-speed PCA was conducted on aggregated data from all seven speed conditions. For the recurrent state, the elliptical shape is retained, whereas a ‘figure-eight’-shaped projection arises in the actuation trajectory. At faster walking speeds, both the recurrent and actuation states seem to scale or ‘stretch’, generating a speed-dependent separation of trajectories.

We employ neural ablations in order to investigate the causal links within the neural network controller, similar to (Merel et al., 2020). Ablation involves latching the activation of target neurons to their cycle-average neural activation during normal walking. For the recurrent state, this in effect, forces the neural state to the center of the typical limit cycle trajectory shown in Figure 5. We apply neural ablations at the start of the trial, and keep those neurons ablated throughout the entirety of the agent’s response to lateral perturbation.

### 2.9 Computing relative contribution of upstream neural activity driving downstream actuation behavior

We quantify the degree to which upstream neurons drive downstream actuation by leveraging the fact that neural network models are backward-differentiable and internal gradients can be computed. Similar to the gradient-times-input methodology proposed in Layer-wise Relevance Propagation, we can estimate the relative contribution from different inputs to the output actuation (Bach et al., 2015; Samek et al., 2015). We can do this for different inputs, such as sensory signals as well as command signals. We can also extend this to compute the relative contribution of internal recurrent neurons to output actuation. For example, to obtain the relative impact of upstream neurons to a specific joint actuation behavior, we first compute the gradients of the actuation activity with respect to the upstream recurrent neurons of interest. We then compute the product of these gradients and the corresponding upstream recurrent neural activation. To implement this in PyTorch, we set the flag requires_grad=True() for the upstream recurrent neural states and we compute the gradients with respect to the downstream output actuation neuron using backward().

## 3 Results

### 3.1 Training and evaluating locomotion policies for robustness

To achieve our goal of analyzing and interpreting robust quadrupedal locomotion policies, we first define a quantitative metric for robustness, and second, train policies that perform well relative to our defined robustness metric. In this work, we focus on the agent’s ability to recover from physical disturbances, specifically a surprise lateral external force during forward walking on flat ground. We estimate robustness by simulating, in parallel, batches of agents exposed to lateral disturbances, and recording recovery rates, defined as the percentage of agents that successfully recover and continue walking. Our experiments consist of many batches, grouped by policy, disturbance magnitude, and timing within the gait cycle when the disturbances are applied.

**FIGURE 2:**
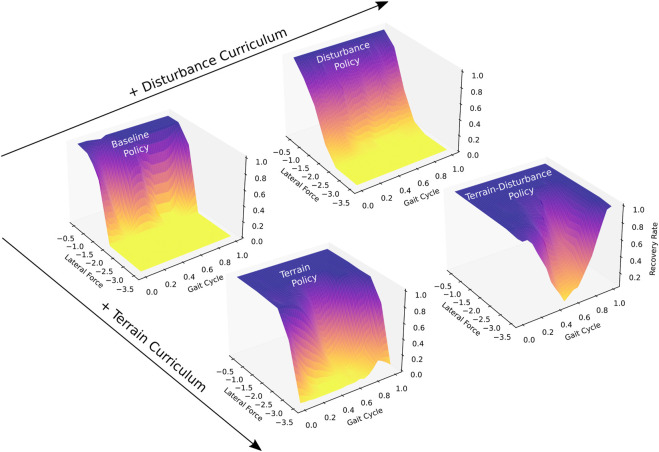
Recovery rates to lateral perturbations, of four quadrupedal control policies trained with different curricula (Baseline, Terrain, Disturbance, Terrain-Disturbance), which serve as a measure of stability. Recovery rates vary based on the magnitude of the lateral force as well as the time during the gait cycle when the perturbation is applied. Disturbances are applied for 80 ms duration, and their magnitudes are scaled by body weight. The beginning of the gait cycle is instantiated after the front left and right hind feet enter stance, touching down on the ground. Agents become more stable when trained with terrain curriculum, and become even more stable when interleaved with a disturbance curriculum. Recovery rate is computed as the ratio of agents that recover from disturbance, relative to all agents (*N* = 100).

We obtain a Baseline policy by training agents on flat ground. Following intuition, Figure 2 shows that gait-averaged recovery rates approach 100% for trials where lateral external forces approach zero, and monotonically decrease as external forces increase in magnitude. The Baseline policy achieves a gait-averaged recovery rate of 99.7% for lateral forces of 0.5 times body weight, however this falls to 0.05% when lateral forces are increased to 2.0 timed body weight.

We next obtain a Terrain policy by training agents on mixed terrain, described in Section 2.2. The Terrain policy is significantly more robust than the Baseline policy, achieving a mean recovery rate of 99.7% for lateral forces up to 1.5 times body weight. When disturbance magnitudes are increased to 2.0 times body weight, the Terrain policy maintains a relatively high gait-averaged recovery rate of 91.0%.

We introduce random disturbances during training of the Baseline and Terrain policies to generate Disturbance and Terrain-Disturbance policies. Upon comparing the gait-averaged recovery rates, we find that adding random disturbances during training result in significantly higher recovery rates for the Terrain-Disturbance policy, relative to the Terrain policy. For a 3 times body weight disturbance, the mean recovery rate for the Terrain-Disturbance policy is 76.1%, meanwhile it is only 21.0% for the Terrain policy. In contrast, the benefits of introducing random disturbances during training are not seen in the Disturbance policy. The gait-averaged recovery rates are similar, if not slightly lower, for the Disturbance policy relative to the Baseline policy.

Recovery rates depend not only on the control policy and the disturbance magnitude, but also the part of the gait cycle that the agent is in when the disturbance is applied. We define the beginning of the gait cycle as the time after both the left front and right hind feet make contact with the ground. Figure 2 captures this gait-dependent robustness, with Terrain and Terrain-Disturbance policies exhibiting significantly lower recovery rates in the middle of the gait cycle. For example, recovery rates of the Terrain-Disturbance policy are 98% when disturbances are applied at the beginning of the gait cycle, and drop to 5% when disturbances are applied in the middle of the gait cycle 200 ms later.

When lateral disturbances are increased to 3.5 times body weight, we see that the Terrain-Disturbance policy is capable of recovering from lateral disturbances, with a 98% recovery rate when disturbed at the start of the gait cycle. The Terrain-Disturbance policy is clearly the most robust when faced with 3.5 times body weight disturbances, with a gait-averaged recovery rate of 54.1%, compared to 3.6% with the Terrain policy and 0% with the Baseline and Disturbance policies.

### 3.2 Bio-mechanical behavior of robust legged robots

Now that we know the Terrain and Terrain-Disturbance policies are more robust to physical perturbations than the Baseline and Disturbance policies, we study their bio-mechanical responses as a way to deepen our understanding of how agents robustly recover from lateral disturbances.

**FIGURE 3:**
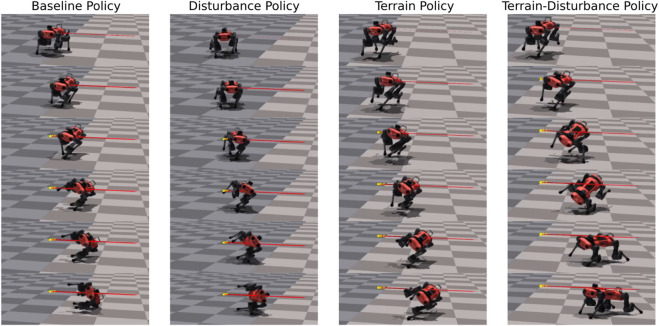
Time lapse of four agents trained with different curricula (Baseline, Disturbance, Terrain, Terrain-Disturbance), after applying a 3.5 times body weight disturbance. The agile RH hip reflex unique to the Terrain-Disturbance policy is visible in the second and third snapshot on the right.

In Figure 3 and in the supplementary video, we provide a comparative visualization of single trial lateral disturbance tests, with agents trained via the Baseline, Terrain, Disturbance, and Terrain-Disturbance policies. Visual inspection of these catalogued snapshots reveal the Terrain-Disturbance agent regains balance, and before doing so, its right hind (RH) leg rapidly swings out to the right before its foot makes contact with the ground. This distinct behavioral response is not apparent in agents trained by the other three policies, which all fail to recover.

**FIGURE 4:**
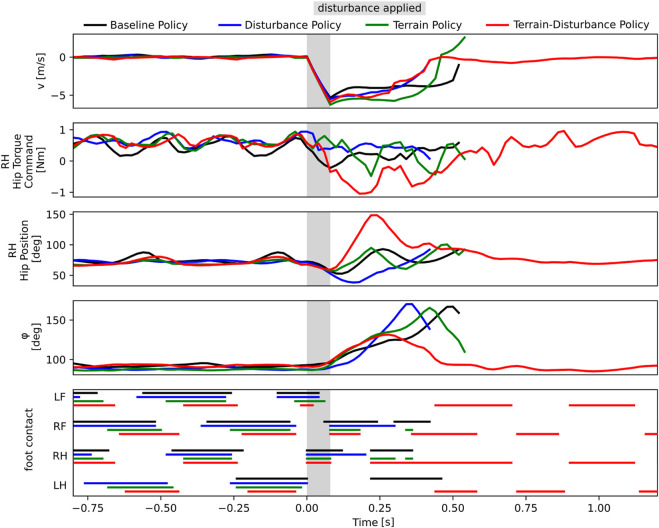
Time history of bio-mechanics data from four agents trained with different curricula (Baseline, Dist, Terrain, Terrain-Disturbance), where a lateral disturbance of magnitude 3.5 times body weight and duration 80 ms is applied. Only the robot with Terrain-Disturbance control policy is stable enough to recover from the disturbance. This agent reacts with a large torque command in the RH hip joint, enabling a wider hip position and wider stance during recovery. The agent trained with Terrain curriculum is able to catch itself in a similar manner, but it does not reach as wide of an angle. The timing of ground-foot contact is shown for left front (LF), right front (RF), right hind (RH), and left hind (LH) legs.

In order to quantitatively measure this phenomenon, we study and compare the bio-mechanical data each of the four control policies. Since agents sideslip and roll when laterally perturbed, we visualize the time series recordings of the lateral body velocity *v* and the roll angle *ϕ* in Figure 4. Additionally, since snapshots in Figure 3 reveals the Terrain-Disturbance agent responding with rapid RH hip joint movement, we also visualize the RH hip position and corresponding torque command in Figure 4. This data reveals that the peak RH hip torque of the Terrain-Disturbance control policy is significantly larger than the other three policies, which in turn leads to a much wider RH hip angle and foot position. The Terrain-Disturbance RH hip angle is able to reach 150°, allowing the leg to swing out further, moving the center of pressure further to the right of the center of mass, and generating greater traction force to stabilize the agent.

The Terrain agent also successfully gets its RH foot onto the ground after the perturbation, however it does not maintain ground contact, and the agent’s roll orientation continues to increase until it falls on its side. This failure may be driven by the fact that the hip angle stops around 100°, prevent the agent from achieving a wider stance and generating sufficient traction.

We also visualize the gait patterns of the four different controllers in Figure 4, and find that during normal walking, different controllers exhibit different gaits. The Baseline and Disturbance controllers have longer stance periods and shorter stride periods, resulting in sometimes three or four feet being in contact with the ground. The Terrain policy has shorter stance periods, but still exhibits some gait pattern asymmetry. The Terrain-Disturbance policy more consistently exhibits two legs in stance at any given time, and sometimes even just a single leg in stance.

### 3.3 Neural dynamics during unperturbed and slightly perturbed walking

So far, we have identified that robust agents exhibit distinct behavioral responses to disturbances, based on the bio-mechanical analysis presented in Section 3.2. Here, we study how neural patterns are implicated into locomotion, by shifting our focus from bio-mechanics to sensory, recurrent, and actuation neural activity of the agent. In this section, we begin by first studying the nominal case of unperturbed locomotion, and then examine walking in the presence of low-magnitude neural and physical perturbations.

One challenge in studying neural activity, is that often the neural populations of interest are high dimensional. In our case, there are 176 sensory neurons, 256 recurrent neurons and 12 actuation neurons. To address this, we perform principal component analysis (PCA), a dimensionality reduction method described in Section 2.6. After the data is z-score normalized and projected into PCA space, we are able to visualize the data along the highest-varying principal component (PC) directions. Section 2.6 describes this process in further detail.

We conduct a set of trials with forward walking speed command u* varying between 0.8 and 2.0 m/s. We independently perform PCA and cycle averaging for observation, recurrent, and actuation states, and produce the neural activity shown in Figure 5. The recurrent state trajectories maintain their shape as walking speed is modulated, but appear to vary in scale. The actuation neural state also shows increased scaling at faster walking speeds. This leads to separation between trajectories of different speeds, with no noticeable areas of overlap when projected into the top three PC directions. Sensory observation states increase in scale and translation with faster walking speeds. We also observe that gait cycles shorten for faster walking speeds, i.e., faster walking leads to faster gait cycles. Gait cycle frequency is roughly 1.8 Hz when walking forward at 0.8 m/s, and 2.3 Hz when walking at 2.0 m/s, which is roughly a 28% increase in mean trajectory speed. We find similar patterns when varying *v* and *r* as well.

**FIGURE 6:**
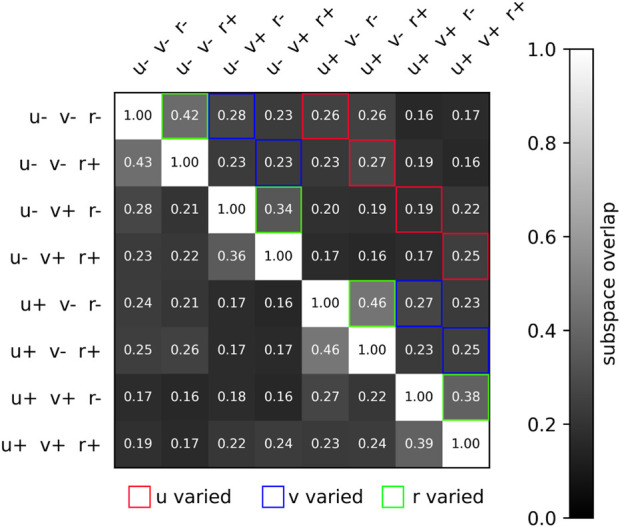
Two-dimensional matrix illustrating the subspace overlap of different velocity conditions. Specifically, the eight conditions are the 2^3^ combinations of u* = ±2.0 m/s, v* = ±2.0 m/s, *s*, r*±0.25 ^rad^/_*s*_. The subspace overlap is greatest when *r* is varied, significantly lower when *v* is varied, and lowest when *u* is varied. This conforms to our intuition, that turning while walking is requires only a slight adjustment, whereas walking forward and walking sideways requires significantly different gait patterns. Note that we are only transforming data using the first 10 PCs, so the *ij* and *ji* subspace overlap values vary slightly.

In addition to visually studying the neural separation across trials, we can quantify the subspace overlap (Russo et al., 2020) across any two trials, as defined in Section 2.7. This pairwise metric measures the degree to which the neural activity of two trials overlap within a common subspace. We find that neural trajectories are relatively similar when yaw rate commands are inverted from positive to negative, whereas the difference is greater when the forward speed command is inverted, or when the lateral speed command is inverted. This is expected since flipping yaw rate direction involves minor gait adjustments, whereas flipping forward speed or lateral speed implicates joint torques in very different ways. This is illustrated in Figure 6, where the subspace overlap between different conditions is shown. We see that the subspace overlap is unity along the diagonal when comparing neural datasets to themselves. Subspace overlap is higher when *r* is inverted, with a mean subspace overlap of 0.40 than when *u* or *v* is inverted, which have mean subspace overlaps of 0.24 and 0.26. And for inversions of a single velocity command, the subspace overlap is generally higher than when two or all three velocity commands are inverted.

We also want to study the agent’s neural dynamics, which we accomplish through conducting two perturbation-based experiments. The first experiment involves three low-magnitude targeted neural perturbations. The second involves a small physical perturbation.

**FIGURE 7:**
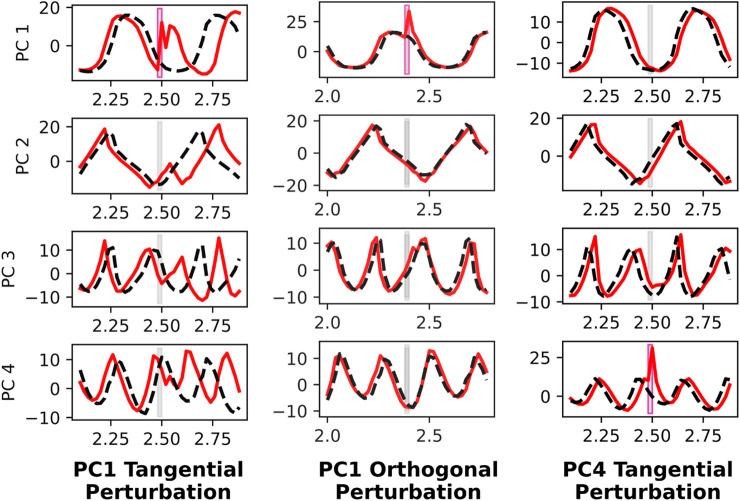
Neural perturbation response of recurrent states in PC1 through PC4 directions, before and after targeted neural perturbations. A perturbation is applied to PC1 that is tangential to the PC1-PC2 plane (left), which causes a significant phase shift in the gait cycle. This is evident in PC1 through PC4. However, the same perturbation applied later such that it is orthogonal to the PC1-PC2 trajectory (middle), does not cause any disruption to the gait cycle. When applying a perturbation in the PC4 direction (right), there is little impact on the population activity, despite it being tangential to PC1-PC4 movement.

To understand the neural dynamics during quadrupedal robot walking, we apply targeted neural perturbations during forward 2.0 m/s walking. Since there are 256 recurrent neurons, and some are implicated more than others in forward walking, we choose to apply neural perturbations that are in the top principal component directions. Specifically, we conduct three trials. The first trial applies a neural perturbation in the first principal component direction, and is timed such that the perturbation is tangential to the direction of neural movement within the ellipsoidal trajectory in the PC1-PC2 space. The agent’s neural response presented in the left column of Figure 7 is salient, resulting in a persistent phase offset visible in neural activity spanning PC1 through PC4. In contrast, when the same neural perturbation is applied at a different time instant, such that the perturbation is orthogonal to the PC1-PC2 neural movement, PC1 neural activity asymptotically converges back to the nominal trajectory and PC2 through PC4 activity is relatively unaffected. The third and final trial involves a neural perturbation in the PC4 direction, and this does not impact neural activity spanning PC1 through PC4. These results indicate that the neural activity is more greatly affected when perturbed in the PC1 direction than the PC4 direction, and when the perturbation is tangential to the direction of neural movement than then it is orthogonal. In all three trials of neural perturbations, the agent recovers.

**FIGURE 8:**
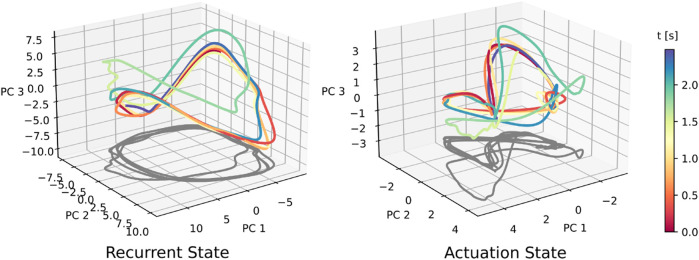
Perturbation study of agent while walking forward at 2.0 m/s reveals (left) the recurrent state and (right) the actuation state move in a direction orthogonal to its direction of motion and ultimately converge back to their nominal trajectory within nearly one cycle. Specifically, at *t* = 1.5 s the perturbation is applied for a single time step, after which the recurrent and actuation trajectories experience transient responses. This is a single-agent trial (*N* = 1), so there is no across-trial averaging.

In the second experiment, we perturbed the agent during forward 2.0 m/s walking with a random change in linear body velocity. Again, we are interested in how the neural activity is affected. When the agent receives its virtual ‘push,’ its recurrent and actuation states are perturbed off their nominal trajectory, as shown in Figure 8. Within one to two gait cycles, the recurrent and actuation state converges back to their nominal cyclic trajectory, as the agent regains its balance.

### 3.4 Structure-based neural ablations

To deepen our understanding of the mechanisms that drive robust behavior, we observe the effect of performing neural ablations. A convenient starting place is to ablate neurons based on their structure. For example, sensory neurons, or observations, are structured in the order shown in Figure 1. To ablate these sensory neurons, we override them to their mean neural activation. We perform two sets of experiments.

In the first experiment, we observe how forward walking is affected when specific sensory neurons are ablated. We observe that the agent behavior changes during forward walking, depending on which sensory neurons are ablated. Ablating *u* causes the agent to walk faster, ablating *v* cause greater sideslip, and ablating the *z*-direction body-frame gravity vector causes greater yawing. Ablating the sensory signals *w*, *p*, *q*, as well as orientation signals *sin*(*θ*) and *sin*(*ϕ*) have no visible effect on forward walking. These results are catalogued in the second column of Table 2.

**TABLE 2:** Tabulated results of normal walking trials and disturbance trials during which structure-based neural ablations are made. The first experiment is summarized by the middle column, which catalogues behavioral changes observed when various sensory neurons are ablated during normal walking. The second experiment is summarized by the rightmost column, which lists recovery rates when specific sensory neurons are ablated simultaneously with a lateral perturbation of 3.5 times body weight. Recall from Section 3.1, that the recovery rate for a 3.5 times body weight lateral disturbance with no ablations is 1.00.

Neural ablations	Normal walking trial	Disturbance trial
	Behavior	Recovery rate
*u* ← 0	|*u*|*↑*	0.78
*v* ← 0	|*v*|*↑*	0.00
*w* ← 0		0.75
*p* ← 0		0.12
*q* ← 0		0.99
*r* ← 0		1.00
*sin*(*θ*) ← 0		0.98
*sin*(*ϕ*) ← 0		0.55
- $\sqrt{1-sin^{2}\left(\theta \right)-sin^{2}\left(\phi \right)}\leftarrow -1$	|*ψ*|*↑*	1.00
u* ← 0	*u* = 0	0.68
v* ← 0		1.00
r* ← 0		1.00
$\theta_{\text{joint}}\leftarrow \bar{\theta_{\text{joint}}}$		0.00
$\omega_{\text{joint}}\leftarrow \bar{\omega_{\text{joint}}}$		0.79
$d\leftarrow \bar{d}$		0.60

In our second experiment, we apply a lateral perturbation to agents while simultaneously ablating targeted sensory neurons, essentially removing sensory feedback from the agent’s disturbance response. Recall from Figure 2 that the nominal recovery rate for Terrain-Disturbance is 100% when no neurons are ablated. We observe that the recovery rates to lateral disturbances vary, depending on which sensory neurons are ablated. For example, the ablation of sideslip *v*, roll rate *p*, and roll signal *sin*(*ϕ*) cause the largest decreases in recovery rates.

In contrast to the sensory and command input neurons, recurrent neurons do not have an explicit structure. Because the initial weights of the recurrent neural networks are randomized before training, we do not know which recurrent neurons, if any, are driving recovery behavior. However, one structure-based ablation that is possible, is to simply ablate all recurrent neurons. When we ablate all the recurrent neurons, agents are still able to walk, albeit at a slower speed of 1.6 m/s. This indicates that recurrent neurons are implicated in forward walking, but are not necessary. The sensory feedback signals and their direct feedforward pathway through the LSTM network alone enable forward walking.

### 3.5 Gradient-based neural ablations

In this section, we study the causal relationships between neural and physical behavior by exploiting the fact that artificial neural networks are backward-differentiable. We interrogate the neural network controller with the aim of identifying the specific upstream neurons that drive specific behavioral responses.

From the bio-mechanical analyses in Section 3.2, we have observed that RH hip actuation is a part of the robust recovery response. Based on this knowledge, we analyze upstream neurons in an effort to identify particular neurons that drive the rapid actuation of the RH hip. We focus on three regions of the neural network, the sensory and command neurons that are input to the LSTM network, the recurrent neurons that are input into the LSTM network, and the recurrent neurons that are outputted by the LSTM network.

**FIGURE 9:**
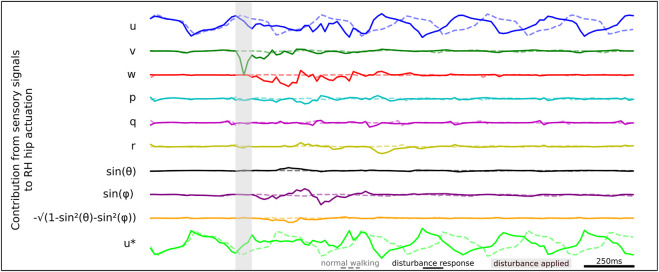
Relative contributions of different sensory neurons to the RH hip actuation response during lateral perturbation recovery.

We apply the gradient-times-input methodology to estimate the contribution of each sensory signal to RH hip actuation, as illustrated by the time series data shown in Figure 9. When studying the sensory states that drive actuation, we identify a series of sensory neurons that drive RH hip actuation at different times during the recovery. These findings are consistent with our ablation study results previously presented in Table 2, where we find significant degradation of agent robustness during perturbation. We observe a strong signal from *v* which contributes to driving the initial RH hip torque actuation. We observe that later in the disturbance response, the roll signal *sin*(*ϕ*) also contributes to RH hip torque actuation.

We also compute the gradient-times-input for recurrent neurons outputted by the LSTM network. We compare these signals during lateral disturbance to the nominal activity seen during a undisturbed gait cycle. For hidden recurrent states outputted by the LSTM network, which feed into actuation, we find that hidden neurons 13, 56, 101, and 108 exhibit the greatest deviation. We confirm the causal relationship between neural activity and behavior by ablating these four neurons and observing robust recovery behavior drops from 100% to 42% for a 3.5 times body weight lateral perturbation.

We perform the same computation in an attempt to identify recurrent neurons inputted to the LSTM network that exhibit a large gradient-times-input deviation. However, when ablated, recovery rates are not affected to the same degree as seen with the output recurrent neurons. This is likely due to the fact that the magnitude of the gradient-times-input deviation is significantly lower for input than output recurrent neurons. To further examine how recurrent neurons inputted to the LSTM drive robust behavior, we look to random ablation methods.

### 3.6 Random sampling-based neural ablations

More broadly, we study the impact of random neural ablations to locomotion robustness as a means of identifying key neurons and the behaviors they drive. This work is inspired by computational neuroscience experiments that ablate individual neurons within a population to understand the neural basis for behavior. In some neural systems, experiments such as these can elicit meaningful conclusions, such as identifying key command neurons (Hampel et al., 2015; Zhang and Simpson, 2022). However, it can also become intractable when neural populations become large, or ineffective when behaviors are driven by more than a single neuron. With artificial neural networks, evaluation can be orders-of-magnitude faster with tensor-accelerated processing, however combinatorial explosion still limits complete evaluation. Therefore, it is necessary to employ sampling strategies in order to make these ablation studies feasible.

**FIGURE 10:**
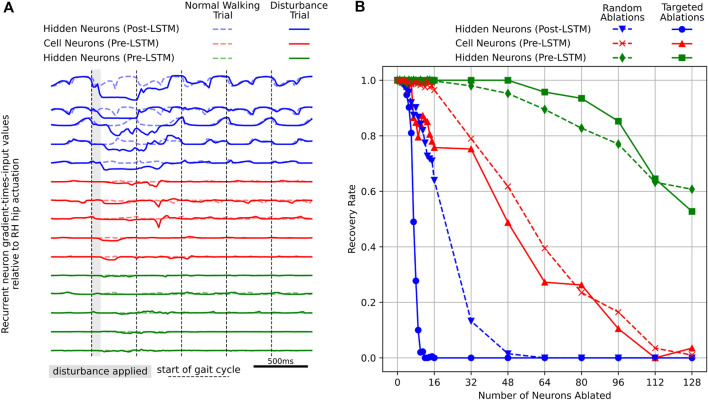
On the left **(A)**, top five targeted post-LSTM hidden neurons, pre-LSTM cell neurons, and pre-LSTM hidden neurons. Targeted neuron ablations are ordered from largest to smallest deviation of gradient-times-input values relative to RH hip actuation. Note that this deviation is measured across the first gait cycle following the lateral disturbance, and is relative to nominal neural activity during normal walking. On the right **(B)**, recovery rates for varying numbers of targeted and random neural ablations. Recovery rates are most sensitive to targeted ablation of output, or post-LSTM hidden neurons. These recurrent neurons also exhibit the largest gradient-times-input deviations, indicating they help drive the rapid RH hip actuation behavior after lateral disturbances.

Here, we conduct trials where agents are laterally disturbed while random recurrent neurons are simultaneously ablated. For example, we perform 400 trials in parallel, and in each trial, a random set of eight recurrent neurons is ablated. We then perform another 400 trials, where we ablate those same sets of neurons, in addition to new random sets of eight recurrent neurons. We continue repeating these trials until all 128 neurons are ablated. We perform these experiments for output (post-LSTM) hidden recurrent neurons, as well as input (pre-LSTM) hidden and cell recurrent neurons. We observe that as the number of randomly-ablated neurons increases, recovery rates decrease, as shown in Figure 10B. We also find that recovery rates are most sensitive to ablation of post-LSTM hidden neurons, somewhat sensitive to ablation of pre-LSTM cell neurons, and least sensitive to ablation of pre-LSTM hidden neurons. Additionally, we repeat all trials by performing targeted ablations, where the neuron ablations are ordered from greatest gradient-times-input to least. We find that targeted ablations of post-LSTM hidden neurons significant lowers recovery rates, relative to random ablation trials. This phenomenon is weaker for pre-LSTM cell recurrent neurons, and in fact inverted for pre-LSTM hidden neurons. The lower gradient-times-input values shown in Figure 10A may be the reason why the targeted ablations of input recurrent neurons does not result in greatly lower recovery rates than random ablations. These neurons simply are not very implicated in driving RH hip actuation.

**FIGURE 11:**
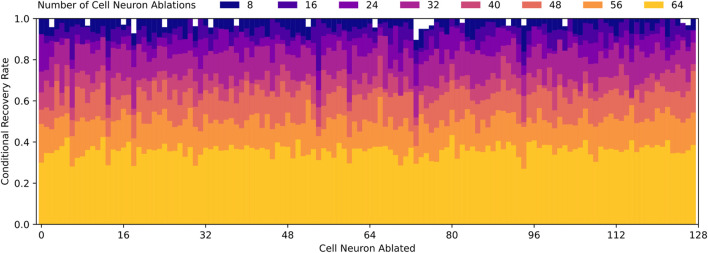
Conditional recovery rates for varying number of random cell neuron ablations, which is defined as the ratio of successful recovery trials to total trials where a specific cell neuron is ablated relative. Recovery rates decrease as the number of cell neuron ablations increase, which aligns with Figure 10. Note that conditional recovery rates are relatively similar regardless of which cell neurons are ablated, with the exception of cell neurons 6, 13, 18, 54, 60, 73, and 94. These specific neurons experience lower conditional recovery rates, which suggest they may play a more significant role in robust recovery behavior than the average cell neuron.

Based on Figure 10B, it is clear that pre-LSTM cell neurons contribute to the robust recovery behavior, however, it is still unknown which neurons are most significant. In order to identify these neurons, we look to the data from each random ablation trial. We compute the conditional recovery rate, based on whether a specific neuron is ablated or not. If all neurons contribute equally to the robust recovery response, we would expect conditional recovery rates to be equal across all neuron ablations. However, the actual conditional recovery rates do vary based on if particular neurons are ablated, as displayed in Figure 11.

From this data, we quickly identify that pre-LSTM cell neurons 6, 13, 18, 54, 60, 73, 94 are much more often implicated in failed recoveries than the average neuron. To confirm the significance, we ablate these seven neurons and find that the recovery rate drops from 100% to 3%. This is significant because random and targeted ablation trials, as depicted in Figure 10, do not approach such a low recovery rate until nearly all 128 pre-LSTM cell neurons are ablated.

## 4 Discussion

### 4.1 Robust bio-mechanical recovery response relies on stepping strategy

Through bio-mechanical analysis, we found that the most robust agents agents, trained via the Terrain-Disturbance policy, exhibit rapid RH hip actuation when responding to a surprise lateral disturbance. To understand why this agile response arises, we look at disturbance-based legged locomotion studies of insects (Jindrich and Full, 2002; Revzen et al., 2013), humans Hof et al. (2010), and robots (Kasaei et al., 2023). In (Hof et al., 2010), it is concluded that humans recover from lateral perturbations by taking a wider next step and also shifting their center of pressure through ankle adjustment. Our quadrupedal robots do not have ankle joints, which leaves the stepping strategy as the most accessible option. This corroborates with our bio-mechanical study, which reveals the RH hip actuation enables the agent to take a wider step and regain balance Additionally, we find agents trained with this policy often has fewer legs in stance, which may increase ability to produce a more agile response.

This recovery behavior is likely learned through the terrain curriculum presented during training. This is because the Terrain-Disturbance policy only experiences disturbances of 0.24 times body weight in training, yet is able to recover from 3.5 times body weight disturbances during robustness trials. This is something that the Disturbance policy cannot do. This suggests that during training, Terrain and Terrain-Disturbance agents are not directly learning how to reject external disturbances, but are learning how to recover after losing balance, something that happens often when agents are learning to traverse mixed terrain. Conversely, the Baseline and Disturbance agents are much less likely to lose balance during training, since they are not challenged to walk on mixed terrain, and simply learn to walk on flat terrain.

### 4.2 Analogies between biological and artificial recurrent neural networks

The cyclic neural activity visualized in Figure 5 agrees with similar reports of cyclic neural activity during bipedal (Siekmann et al., 2020) and quadrupedal robot (Chiappa et al., 2022) locomotion tasks. Additionally, rapid convergence to the nominal elliptical trajectory after perturbation shown in 8 suggests it is a stable limit cycle.

Additionally, our analysis confirms that altering the agent walking speed and gait speed, leads to the recurrent neural activity moving to different parts of the neural state space. Prior work demonstrates that this phenomenon occurs in recurrent neural networks, regardless of the task (Sussillo and Barak, 2013; Maheswaranathan et al., 2019b), because in recurrently-driven systems, altering trajectory speed, in this case gait speed, requires moving to a different region of state space (Remington et al., 2018). The patterns of activity presented here are also similar to the trajectory separation found in primate cycling tasks (Saxena et al., 2022).

Based on the data shown in Figure 7, we find that perturbing neurons along dominant PC directions elicits a larger magnitude response, and also implicate other neural populations as well. Perturbation responses return to nominal activity within less than half of a gait cycle. Additionally, neural perturbations cause a phase shift in the gait cycle when perturbations are tangential to the instantaneous direction of neural activity, causing greater interaction with the neural dynamics. In contrast, when perturbations are orthogonal to the direction of neural activity, the effect on the network is nearly negligible. These two findings suggest that our RNN-based controller exhibits structured low-dimensional neural dynamics, similar to primates (O’Shea et al., 2022).

### 4.3 Command neurons and sensory feedback drive locomotion

From biology, we know commands signals from higher-level parts of the nervous system typically control animal behavior (Hampel et al., 2015; Zhang and Simpson, 2022). Autonomous systems often vary in their structure, with some deep RL controllers learning end-to-end navigation and locomotion control, and others separating these into two different modules. In this work, our learned controller solely performs low-level locomotion control, and adjusts its behavior based on receiving external user-defined velocity commands, (u*, v*, r*).

We find that forward walking behavior is driven largely by the forward velocity command and forward velocity sensory feedback signal. Ablating forward velocity *u* causes the agent to walk faster. Despite the controller not having explicit control laws, it appears that ablating the sensory input *u* = 0, when actually *u* > 0, causes a positive feedback and drives *u* higher and higher until agents fall over from locomoting too fast. Additionally, we find that robust recovery behavior is also driven by sensory feedback. Ablating sensory neurons, especially signals that are activated during lateral disturbances, greatly reduce robustness.

### 4.4 Model and sampling-based ablations generate neural hypothesis

Computing model gradients has been shown to be an effective means of identifying which output, or post-LSTM hidden recurrent neurons drive RH hip actuation. Targeted ablation studies have provided confirmation that these neurons are necessary for robust recovery.

We applied the same methodology for targeted ablations of input, or pre-LSTM hidden and cell neurons, despite them having significantly lower gradient-times-input values. The result is that recovery rates are not significantly different between targeted and random ablations. However, we do see recovery rates degrade as more neurons are ablated, suggesting some of these neurons that are required for a robust response, despite the fact that they are not driving the RH hip actuation behavior.

Large-scale sampling-based neural ablations and analysis of successful recovery trials enabled us to identify seven input, or pre-LSTM cell neurons that drive robust behavior. Without them, only 3% of agents successfully recovery from lateral disturbances. Only two of these seven neurons were previously identified through the targeted gradient-based RH hip actuation methodology. The other five neurons do not drive this particular actuation behavior, yet are still significant to the overall robustness of the agent during lateral disturbances.

## 5 Conclusion

This work proposes approaches for elucidating the neural basis for robust legged locomotion. Similar to prior work, we identified and characterized cyclic patterns of neural activity that are inherent to locomotion. We compare controllers trained via different curricula, and found agents trained with terrain and disturbance curricula are the most robust to physical perturbations.

We observe distinct behavioral responses of robust agents, specifically a rapid actuation of the hip joint, which led to a wider stance to regain balance. We examine the gradients within the robust model to identify which neurons drive this specific behavior. Leveraging this model-based method, we identify key output recurrent neurons and and sensory signals that drive this behavior as well. We find that input recurrent neurons are not as implicated in driving the rapid hip joint response, but through a supplemental sampling-based ablation strategy, identify neurons that are are critical to robust response. By interleaving physical perturbations with neural ablations, as well as model information and sampling techniques, we have further elucidated the neural and behavioral bases of robust quadrupedal robot locomotion.

## Data Availability

The original contributions presented in the study are publicly available. This data can be found here: https://github.com/generush/neuro-rl-sandbox.

## Footnotes

1
https://github.com/NVIDIA-Omniverse/IsaacGymEnvs

2
https://github.com/Denys88/rl_games
